# A Genome-Wide Association Study for Calving Interval in Holstein Dairy Cows Using Weighted Single-Step Genomic BLUP Approach

**DOI:** 10.3390/ani10030500

**Published:** 2020-03-17

**Authors:** Hadi Atashi, Mazdak Salavati, Jenne De Koster, Mark A. Crowe, Geert Opsomer, Miel Hostens, The GplusE Consortium

**Affiliations:** 1 Department of Reproduction, Obstetrics and Herd Health, Ghent University, Merelbeke 9820, Belgium; atashi@shirazu.ac.ir (H.A.); jenne.dekoster@zoetis.com (J.D.K.); Geert.Opsomer@ugent.be (G.O.); 2 Department of Animal Science, Shiraz University, Shiraz 71441, Iran; 3 The Roslin Institute and Royal (Dick) School of Veterinary Studies, University of Edinburgh, Easter Bush, Midlothian EH25 9RG, UK; Mazdak.salavati@roslin.ed.ac.uk; 4 School of Veterinary Medicine, University College Dublin, Belfield, Dublin D04 V1W8, Ireland; mark.crowe@ucd.ie; 5 Department of Farm Animal Health, University of Utrecht, Yalelaan 7, 3584 CL Utrecht, The Netherlands

**Keywords:** genome-wide association study, Holstein, calving interval

## Abstract

**Simple Summary:**

Reproductive performance is an important factor, which determines productive life and drives culling decisions in dairy herds. There are strong motives for including reproductive performance in genetic selection programs of dairy cows; however, low heritability estimates reported for reproductive performance measures limit the genetic selection efficiency. More effective genetic selection could be achieved using genomic information. The aim of this study was to identify genomic region(s) associated with the length of the calving interval in Holstein cows. The accuracies of genomic estimated breeding values (GEBVs) with single-step genomic BLUP (ssGBLUP) and the pedigree-based BLUP were compared as well. The results showed that the accuracies of GEBVs using the single-step genomic BLUP were much higher than those estimated using the pedigree-based BLUP. We identified three genomic regions (BTA3, BTA6, and BTA7) associated with the length of the calving interval in Holstein dairy cows.

**Abstract:**

The aim of the present study was to identify genomic region(s) associated with the length of the calving interval in primiparous (n = 6866) and multiparous (n = 5071) Holstein cows. The single nucleotide polymorphism (SNP) solutions were estimated using a weighted single-step genomic best linear unbiased prediction (WssGBLUP) approach and imputed high-density panel (777 k) genotypes. The effects of markers and the genomic estimated breeding values (GEBV) of the animals were obtained by five iterations of WssGBLUP. The results showed that the accuracies of GEBVs with WssGBLUP improved by +5.4 to +5.7, (primiparous cows) and +9.4 to +9.7 (multiparous cows) percent points over accuracies from the pedigree-based BLUP. The most accurate genomic evaluation was provided at the second iteration of WssGBLUP, which was used to identify associated genomic regions using a windows-based GWAS procedure. The proportion of additive genetic variance explained by windows of 50 consecutive SNPs (with an average of 165 Kb) was calculated and the region(s) that accounted for equal to or more than 0.20% of the total additive genetic variance were used to search for candidate genes. Three windows of 50 consecutive SNPs (BTA3, BTA6, and BTA7) were identified to be associated with the length of the calving interval in primi- and multiparous cows, while the window with the highest percentage of explained genetic variance was located on BTA3 position 49.42 to 49.52 Mb. There were five genes including *ARHGAP29*, *SEC24D*, *METTL14*, *SLC36A2*, and *SLC36A3* inside the windows associated with the length of the calving interval. The biological process terms including alanine transport, L-alanine transport, proline transport, and glycine transport were identified as the most important terms enriched by the genes inside the identified windows.

## 1. Introduction

Milk yield and reproductive performance are two important factors which determine productive life and culling decisions in dairy herds [1,2]. While improvements in management and nutrition, along with intense genetic selection have increased milk yield in recent decades, reproductive efficiency is among the main causes of culling and replacement of Holstein cows worldwide [3,4,5,6,7]. Norman, et al. [8] reported that despite the continued march of increasing production per cow per year, in the US, the trend for at least some reproductive parameters dairy cows has been improved. It is well documented that declining fertility cannot be improved through improving management alone, hence genetic selection has attracted much attention [9]. Indicators including age at first calving, days from calving to first breeding, days open, the length of the calving interval, pregnancy rates, and the number of services per conception can be used to evaluate the reproductive performance in dairy cows [10,11,12]. Although studies have shown the existence of genetic variance for reproductive performance, the additive genetic variance reported accounted for only a small fraction of the total variance [13,14,15]. There are strong motives for including reproductive performance in genetic selection programs of dairy cows, but the very low heritability estimates reported for most of the considered reproductive performance measures makes selection for reproductive performance not effective enough [9,16]. However, low heritability estimates for reproductive performance does not indicate the unimportance of genetic selection. More effective genetic selection for female fertility could be achieved by using information from the whole genome and incorporating the information of quantitative trait loci (QTL) into selection decisions [9,16]. Genomic information can be used to improve the accuracy of breeding values and to perform genome-wide association studies (GWAS) with the aim of identifying genomic region(s) explaining genetic variance of traits. Although, genome-wide association studies carried out within a variety of cattle breeds identified many single nucleotide polymorphisms (SNPs) associated with the length of the calving interval (CI), as an indicator for reproductive performance, they are mainly based on the polygenic estimated breeding value (EBV), daughter yield deviation (DYD), or deregressed proof for CI [4,17,18,19]. The single-step genomic best linear unbiased prediction (ssGBLUP) approach [20,21], a quite common procedure in GWAS, has the advantage of simultaneously using the phenotypes of genotyped and nongenotyped animals, pedigrees, and genotypes; therefore, there is no need to calculate pseudo-phenotypes. It has been reported that the use of ssGBLUP procedure increased the accuracy of genetic evaluation in many contexts and species compared with pedigree-based BLUP [22,23]. However, the ssGBLUP assumes that all SNPs explain the same amount of genetic variance, which is unlikely in the case of traits whose major genes or QTL are segregating. The weighted single-step genomic BLUP (WssGBLUP) approach [24] allows the use of different weights for SNPs according to their trait-relevant importance and improves the accuracy of genetic evaluation and the precision of estimates of SNP effects . 

The objective of this study was to use the WssGBLUP procedure and imputed high-density panel (777 k) genotypes to identify genomic region(s) associated with calving interval in Holstein dairy cows. The accuracies of genomic estimated breeding values (GEBVs) with the WssGBLUP procedure and the pedigree-based BLUP were compared as well. 

## 2. Materials and Methods 

### 2.1. Phenotypic and Genotypic Data

Data in this study were collected as part of the Genotype plus Environment (GplusE) FP7-Project (http://www.gpluse.eu). The data were records of 11,937 lactations on 6866 primiparous and 5071 multiparous Holstein cows calving between 2010 and 2018, distributed among 118 herds in four countries (Belgium, the Netherlands, Great Britain, and Denmark). The calving interval was calculated as the difference between calving dates from successive parities and was restricted to the range of 270 to 700 d. Genotyping was performed using the Illumina Bovine 10 K low-density chip (n = 20,462), Bovine SNP50K (n = 10,638) or BovineHD SNP panel (795 animals). Genotypes of animals were imputed to high density (HD) with a reference population consisting of 46 HD males and 749 HD females [25]. In total, 12,367 out of 31,895 genotyped individuals, had either phenotypic data or were in the pedigree file which was used in the association analysis. (The number of animals with records was 6866, the number of animals with records and with genotypes was 5345, the number of animals with records and no genotypes was 1521, and the number of animals with genotypes and no records was 7022). Only SNPs located on *Bos taurus* autosomes (BTA) were considered. SNP markers with minor allele frequency less than 5% were excluded. Finally, 566,345 out of 730,539 SNPs were available for the association analysis. Ethics approval and consent to participate were not applicable to this study.

### 2.2. Variance Components Estimation

The pedigree consisted of 43,181 individuals (12,367 and 6866 out of 43,181 animals had genotype and phenotype data, respectively). The genetic analyses were carried out through the average information restricted maximum likelihood (AIREML) via AIREMLf90 from the BLUPF90 software package [26]. A linear single-trait animal model was used for the length of the CI in primiparous cows. The linear model included fixed effect of herd-year-season of calving (HYS), and country, covariate effects of age at first calving in both linear and quadratic forms, as well as animal and residual random effects. The complete model for primiparous cows can be represented as follows:

(1) $$y_{\mathrm{ijk}}=µ+{\mathrm{HYS}}_{i}+{\mathrm{con}}_{j}+b_{1}\left({\mathrm{age}}_{k}\right)+b_{2}{\left({\mathrm{age}}_{k}\right)}^{2}+a_{k}+e_{\mathrm{ijk}}$$

where y_ijk_ represents the length of the CI for animal k, µ is the overall mean, HYS_i_ is the fixed effect of i^th^ herd-year-season of calving, con_j_ is the fixed effect of j^th^ country, b_1_ and b_2_ are the linear and quadratic regression coefficients of the length of the CI on the age at first calving, age_k_ is the age at first calving of k^th^ cows, a_k_ is the additive genetic effect, and e_ijk_ is the random residual error. The additive genetic and residual variances were obtained as follows:

(2) $$\mathrm{var}\left[\begin{array}{c} a\\ e \end{array}\right]=\left[\begin{array}{c} H\sigma_{a}^{2}\;0\\ 0\;I\sigma_{e}^{2} \end{array}\right]$$

where **a** is the vector of direct additive genetic effects, **e** is a vector of residual effects, $\sigma_{a}^{2}$ and $\sigma_{e}^{2}$ are, respectively, total additive genetic and residual variances, and **H** is the genetic relationship matrix combining SNP information and pedigree data (**A**) [20]:

(3) $$H^{-1}=A^{-1}+\left[\begin{array}{c} 0\;0\\ 0\;G^{-1}-A_{22}^{-1} \end{array}\right]$$

where $A^{-1}$ is the inverse of the pedigree-based relationship matrix for all animals; $A_{22}^{-1}$ is the inverse of the numerator relationship matrix for the genotyped individuals; and **G** is the genomic relationship matrix [27].

(4) $$G=\frac{{ZDZ}^{\prime }}{\sum_{i=1}^{M}2p_{i}(1-p_{i})}$$

where **Z** is the matrix of gene content adjusted for allele frequencies (0, 1, or 2 for *aa*, *Aa,* and *AA*, respectively); **D** is a diagonal matrix of weights for SNP variances; M is the number of SNPs, and p_i_ is the estimated minor allele frequency at i^th^ locus. The genetic analyses for the length of the CI in multiparous cows were carried out using a linear single-trait repeatability animal model, which was the same as the model used for primiparous cows but here, the fixed effect of parity was included into the model. In addition, a third random effect representing the environmental permanent effect associated with animals having repeated records was included into the model. This effect, assumed to be uncorrelated with additive genetic effects, allowed for the partitioning of the environmental variance into permanent and temporary components. The complete model for multiparous cows can be represented as follows:

(5) $$y_{\mathrm{ijkl}}=µ+{\mathrm{HYS}}_{i}+{\mathrm{con}}_{j}+p_{k}+b_{1}\left({\mathrm{age}}_{l}\right)+b_{2}{\left({\mathrm{age}}_{l}\right)}^{2}+a_{l}+{\mathrm{pe}}_{l}+e_{\mathrm{ijkl}}$$

Most of the terms in this model were defined as for the linear single-trait animal model except for p_k_, the effect of lactation number, and pe_l_, is the permanent environmental effect of cow l.

Pedigree-based (co)variance components and breeding values were estimated considering the same linear animal model used to estimate the (co)variance components mentioned before. In the pedigree-based BLUP the genomic information was excluded, and the direct additive genetic effects were estimated using the pedigree-based relationship matrix. 

### 2.3. Weighted Single-Step Genome-Wide Association Study

The analyses were performed using the weighted single-step genome-wide association study (WssGWAS) methodology [24], considering the same linear animal model used to estimate the (co)variance components mentioned before. The animal effects were decomposed into those for genotyped (**a_g_**) and ungenotyped animals (**a_n_**). The animal effects of genotyped animals are a function of the SNP effects, $a_{g}=Zu$, where **Z** is a matrix relating genotypes of each locus and **u** is a vector of the SNP marker effect. The variance of animal effects was assumed as follows:

(6) $$\mathrm{Var}\left(a_{g}\right)=\mathrm{Var}\;\left(Zu\right)={ZDZ}^{\prime }\sigma_{u}^{2}=G^{*}\sigma_{a}^{2}$$

where **D** is a diagonal matrix of weights for variances of markers (at iteration 1, SNP weights in the D matrix are equal to 1) and $\sigma_{u}^{2}$ is the genetic additive variance captured by each SNP marker when the weighted relationship matrix (G*) was built with no weight. 

The SNP effects were obtained using following equation:

(7) $$\hat{u}=\lambda {DZ}^{\prime }G^{*-1}{\hat{a}}_{g}={DZ}^{\prime }{\left[Z{DZ}^{\prime }\right]}^{-1}{\hat{a}}_{g}$$

where λ was defined by VanRaden [27] as a normalizing constant, as described below:

(8) $$\lambda =\frac{\sigma_{u}^{2}}{\sigma_{a}^{2}}=\frac{1}{\sum_{i=1}^{M}2p_{i}(1-p_{i})}$$

The following iterative process [24] was used to estimate the SNP effects. Step 1. **D** = **I** in the first step. Step 2. Calculate the **G** matrix. Step 3. Calculate GEBVs for the entire data set using ssGBLUP. Step 4. Estimate SNP effects from solutions of genomic breeding values in the previous step: (û): $\hat{u}=\lambda {DZ}^{\prime }G^{*-1}{\hat{a}}_{g}$. Step 5. Estimate of the effect of each SNP: $d_{i}={\hat{u}}_{i}^{2}2p_{i}\left(1-p_{i}\right)$, where i is the i^th^ SNP. Step 6. Normalize the vector of variances of SNP effects to get the SNP weights (this normalization process ensures that the sum of the variances remain constant and equal to the number of SNP). Step 7. Use SNP weights to construct the **D** matrix; exit or loop to step 2. The effects of markers were obtained by five iterations from Steps 2 to 7. The accuracies of genomic estimated breeding values (GEBVs) with ssGBLUP and the pedigree-based BLUP were estimated using following formula.

(9) $$\mathrm{acc}=\sqrt{1-\frac{\mathrm{PEV}}{\sigma_{g}^{2}}}$$

where **PEV** is the prediction error variance, and $\sigma_{g}^{2}$ is the additive genetic variance of the trait. The percentage of genetic variance explained by i^th^ genomic region was estimated using the following formula.

(10) $$\frac{\mathrm{Var}\left(a_{i}\right)}{\sigma_{a}^{2}}\times 100\%=\frac{\mathrm{Var}\left(\sum_{j=1}^{n}Z_{j}{\hat{u}}_{j}\right)}{\sigma_{a}^{2}}\times 100$$

where $a_{i}$ is the genetic value of the i^th^ region that consists of n consecutive SNP (n = 1, 5, 10, 20, and 50), $\sigma_{a}^{2}$ is the total genetic variance, Zj is the vector of SNP content of the j^th^ SNP for all individuals, and ${\hat{u}}_{j}$ is the marker effect of the j^th^ SNP within the i^th^ genomic region. The results were presented by the proportion of additive genetic variance explained by each genomic region of n consecutive SNP. 

### 2.4. Gene Prospection

The chromosome segments associated with the length of the calving interval were selected to explore and determine potential quantitative trait loci (QTL). The database (version UMD3.1) including gene locations, start positions and end sites for all bovine genes (http://www.ensembl.org/index.html) was used for identification of genes. The list of genes inside the genomic region(s) associated with the length of the calving interval, considered as positional candidate genes, was uploaded to Enrichr for gene ontology (GO) enrichment analysis [28,29]. Significantly enriched biological process terms with at least two genes from the input gene list were identified based on the retrieved adjusted *P* value.

## 3. Results and Discussion

### Variance Components and Accuracy of Genomic Predictions

The mean (SD) of the length of the CI in primi- and multiparous cows were 395.1 (69.1) and 396.7 (62.9) d, respectively. The median of the length of the CI in primi- and multiparous cows were 375 and 381 d, respectively. The additive and residual variances estimated using the AIREML method for the length of the CI in primiparous cows were 201.3 (SE = 57.60) and 3728.7 (SE = 85.76), respectively. The estimated additive, permanent environmental effect, and residual variances in multiparous cows were 198.3 (67.8), 71.4 (100.4), and 2953.7 (103.15), respectively. The heritability estimates for the length of the CI in primi- and multiparous cows were 0.05 (0.01) and 0.06 (0.01), respectively, which are consistent with findings from other studies on dairy cattle [17,30,31,32,33]. Generally, the large unexplained residual variation observed for the length of the CI is attributable not only to the large effect of the environmental factors on this trait but also to the low quality of the data [34].

**Figure 1:**
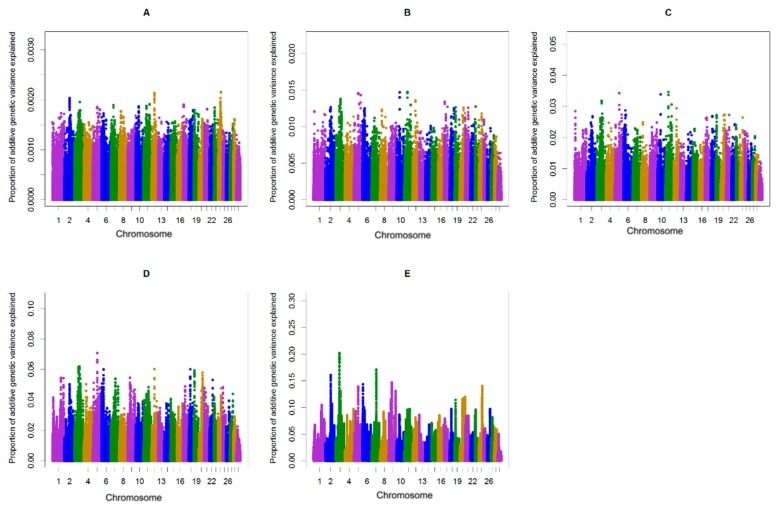
Additive genetic variance explained by windows of (**A**) 1, (**B**) 5, (**C**) 10, (**D**) 20, and (**E**) 50 consecutive single nucleotide polymorphisms (SNPs) across chromosomes for the length of the calving interval in primiparous cows.

**Figure 2:**
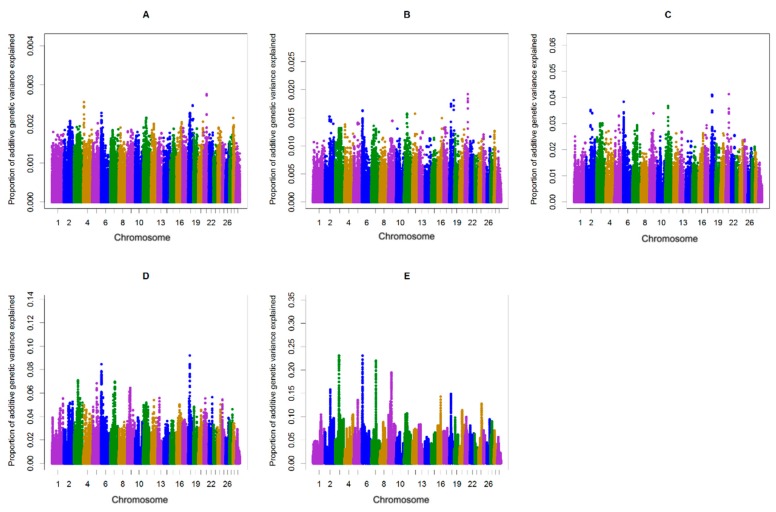
Additive genetic variance explained by windows of (**A**) 1, (**B**) 5, (**C**) 10, (**D**) 20, and (**E**) 50 consecutive SNPs across chromosomes for the length of the calving interval in multiparous cows.

The single-step genomic BLUP (ssGBLUP) method assumes equal variance for all single nucleotide polymorphisms, which is unlikely in the case for traits whose major genes or QTL are segregating. To overcome the limitation of ssGBLUP, unequal variance or weights for all SNP are applied in an approach called weighted single-step genomic BLUP (WssGBLUP), in which SNP effects are weighted according to their importance for the trait of interest [24]. Accuracies of GEBVs estimated for primiparous cows were 0.32, 0.33, 0.32, 0.32, and 0.32 for iterations one to five of WssGBLUP, respectively. The corresponding values for multiparous cows were 0.37, 0.38, 0.37, 0.37, and 0.37. The accuracies of genomic estimated breeding value (GEBV) provided at the second iteration of WssGBLUP improved by +5.4 to +5.7, (primiparous cows) and +9.4 to +9.7 (multiparous cows) percent points over accuracies from the pedigree-based BLUP. In this study, the influence of the number of iterations (1–5) on the accuracy of genomic predictions for the length of the calving interval was investigated. The most accurate genomic evaluation was provided at the second iteration of WssGBLUP, which was used to identify associated genomic regions using a windows-based GWAS procedure. Wang, Misztal, Aguilar, Legarra, and Muir [24] investigated the influence of the number of iterations (1–8) on the accuracy of genomic predictions and showed that the highest accuracies were obtained at the second iteration and then decreased slightly. Previous studies on dairy goats [35,36] and broiler chickens [37] have also reported that the accuracy of GEBVs estimated using WssGBLUP was maximized by the second iteration and then decreased slightly. The decline in accuracy with iteration may be a result of the continuous addition of weights to the SNP with large effects while shrinking the SNP with small influence [38]. In the window-based GWAS procedure, different window types (distinct or sliding windows) and variable window sizes (defined as the number of SNPs or the number of base pairs) can be used. The common form for declaring importance is to use a threshold on the additive genetic variance explained by individual windows [39]. However, it is unclear what window size is optimal and there is no standard to define the threshold on explained genetic variance [39]. Therefore, determining the proper window size is usually subjective and researchers often have not justified their choices or sometimes have acknowledged that their choices are arbitrary [40,41]. In the present study, sliding windows of 1, 5, 10, 20, and 50 consecutive SNPs were used to identify genomic region(s) associated with the length of the calving interval in primi- and multiparous cows and to determine if the region(s) identified may change depending on the window size. General information about the results of ssGWAS for primi- and multiparous cows are described in Tables S1–S5 and Tables S6–S10, respectively. The results of different sliding window sizes in primi- and multiparous cows showed that the identified peaks changed depending on the window size where smaller window sizes (windows of 1, 5, and 10 consecutive SNP) being accompanied with large noises (Figure 1 and Figure 2). Fragomeni, et al. [42] also reported that small window sizes are accompanied with large noises. Furthermore, it has been shown that single-SNP GWAS cannot be effective enough, because single-SNPs provide limited information about the content of flanking genomic regions [43,44,45]. In the present study, 50-adjacent SNP windows (with an average of 165 Kb widow size) that explained equal to or more than 0.20% of the genetic variance was considered as the threshold for significance. Han and Peñagaricano [46] considered 1.5 Mb SNP windows that explained more than 0.50% of the genetic variance as the threshold to declare significance. Suwannasing, et al. [47] using the Porcine SNP60k BeadChip, considered 5-adjacent SNP windows that explained more than 1% of total genetic variance as the threshold to declare significance. de Oliveira Silva, et al. [48] using the BovineHD SNP panel, considered 50-adjacent SNP windows (with average of 280 kb) that explained more than 0.50% of additive genetic variance as the threshold to declare significance. The results identified three windows (on BTA3, BTA6, and BTA7) associated with calving interval in primi- and multiparous cows (Figure 1 and Figure 2). These three regions combined explained 0.51% and 0.68 % of the total genetic variances of the length of the calving interval, respectively, in primi- and multiparous cows (Table 1). The length of the calving interval generally has a low heritability and probably that is the reason why in neither primi- nor multiparous cows windows highly associated with this trait were identified.

Although, genome-wide association studies carried out within a variety of cattle breeds identified many single nucleotide polymorphisms (SNPs) associated with the length of the calving interval (CI), the results are inconsistent [17,18,49]. Zhou, Li, Cai, Liu, Yin, Shi, and Zhang and Zhang [49] using a single SNP regression mixed linear model, identified two SNPs (BTA19 and BAT25) associated with the length of the calving interval in Xinjiang Brown cattle. Minozzi, Nicolazzi, Stella, Biffani, Negrini, Lazzari, Ajmone-Marsan, Williams [17] using a single SNP regression mixed linear model, identified five SNPs (BTA2. BTA5, BTA8, BTA24, and BTA28) associated with the length of the calving interval in Italian Holstein Cattle. Nayeri, Sargolzaei, Abo-Ismail, May, Miller, Schenkel, Moore, and Stothard [18] using a single SNP regression mixed linear model, identified a total of eight highly significant SNPs on BTA21 associated with days open in Canadian dairy Holstein cattle. 

In this study, the genomic region with the highest percentage of explained genetic variance was located on the BTA3 position 49.42 to 49.52 Mb, which overlaps among window sizes of 20 and 50 consecutive SNPs in primi- and multiparous cows. This region also overlaps with QTLs for dystocia [50,51], milk fat yield [52], and residual feed intake [53] in dairy cows. Genes including *ARHGAP29*, *SEC24D*, *METTL14*, *SLC36A2,* and *SLC36A3* were identified inside the windows associated with CI (Table 1). *SLC36A2* was reported to be associated with milk beta-casein percentage, milk kappa-casein percentage, and milk protein yield in Chinese Holstein cows [49]. 

Significantly-enriched biological processes with two genes from the input gene list are shown in Table 2. The biological process including alanine transport, L-alanine transport, and proline transport and glycine transport were identified as the most important term enriched by the identified genes inside the identified windows. Amino acid transport is defined as the directed movement of amino acids, organic acids containing one or more amino substituents, into, out of, or within a cell, or between cells, by means of some agent such as a transporter. An amino acid transporter is a membrane transport protein that transports amino acids. The amino acid transport systems in early embryos likely are regulated at the genetic level by various conditions in the female reproductive tract; however, the precise mechanisms of regulation and their physiological consequences are yet to be fully described [54]. Embryo amino acid content is determined, at least in part, by regulation of amino acid transport [55,56,57]. Lane and Gardner [58] reported that amino acids improve mouse embryo development primarily during cleavage, and support development of more viable embryos. Moore and Bondioli [59] analyzed the bovine oviductal fluid for free amino acid content and reported that glycine and alanine were the two most predominant amino acids. Elhassan, et al. [60] reported that alanine, glutamate, glycine, and taurine, are present in strikingly high concentrations in both bovine oviductal and uterine fluids, suggesting that they might play important roles in early embryo development. Moore and Bondioli [59] reported that glycine and alanine supplementation of culture medium enhances development of in vitro matured and fertilized cattle embryos and concluded that glycine and alanine have a role in early embryonic development. It has been reported that embryo growth and development rates are important indicators of embryo viability and reproductive efficiency [61]. 

## 4. Conclusion

The objective of this study was to identify genomic regions associated with the length of the calving interval in Holstein cows. In this study, the length of the calving interval, a commonly used measure of reproductive performance in dairy cow breeding goals, was used as an indicator for female reproductive performance. We compared pedigree-based BLUP with the WssGBLUP for the length of the calving interval and confirmed that the WssGBLUP method improved the accuracy of GEBV particularly at the second iteration. In the present study, different sliding window sizes were evaluated to identify genomic region(s) associated with the length of the calving interval and the results showed that the identified peaks changed depending on the window size with smaller window sizes being accompanied with large noises. We identified three windows of 50 consecutive SNPs associated with the length of the calving interval. The findings of this study can be used to search for causative mutations, and for breeding through marker-assisted selection to improve the length of the calving interval in Holstein dairy cows.

## Figures and Tables

**Table 1:** Identification of genes based on the additive genetic variance explained by 50-adjacent SNP windows for the length of the calving interval in primi- and multiparous Holstein cows.

Chromosome	Position	Genes ^1^	The Proportion of Genetic Variance Explained in Primi- and Multiparous Cows
BTA3	49426411–49528260	*ARHGAP29*	0.20 and 0.23
BTA6	7734092–7861603	*SEC24D, METTL14*	0.14 and 0.23
BTA7	64522263–64602968	*SLC36A2, SLC36A3*	0.17 and 0.22

^1^ Official gene symbol (assembly UMD_3.1, annotation release 103).

**Table 2:** Gene ontology (GO) terms enriched by the genes inside the chromosomal region of associated milk production and lactation curve parameters.

GO Term Description	Genes
L-alanine transport (GO:0015808)	*SLC36A2*, *SLC36A3*
alanine transport (GO:0032328)	*SLC36A2*, *SLC36A3*
proline transport (GO:0015824)	*SLC36A2*, *SLC36A3*
glycine transport (GO:0015816)	*SLC36A2*, *SLC36A3*

## Supplementary Materials

The following are available online at https://www.mdpi.com/2076-2615/10/3/500/s1. Table S1: Results of 1-adjacent SNP windows in primiparous cows (Primiparous1.xlsx). Table S2: Results of 5-adjacent SNP windows in primiparous cows (Primiparous5.xlsx). Table S3: Results of 10-adjacent SNP windows in primiparous cows (Primiparous10.xlsx). Table S4: Results of 20-adjacent SNP windows in primiparous cows (Primiparous20.xlsx). Table S5: Results of 50-adjacent SNP windows in primiparous cows (Primiparous50.xlsx). Table S6: Results of 1-adjacent SNP windows in multiparous cows (Multiparous1.xlsx). Table S7: Results of 5-adjacent SNP windows in multiparous cows (Multiparous5.xlsx). Table S8: Results of 10-adjacent SNP windows in multiparous cows (Multiparous10.xlsx). Table S9: Results of 20-adjacent SNP windows in multiparous cows (Multiparous20.xlsx). Table S10: Results of 50-adjacent SNP windows in multiparous cows (Multiparous50.xlsx).
